# The Kidney Transplant Evaluation Process in the Elderly: Reasons for Being Turned down and Opportunities to Improve Cost-Effectiveness in a Single Center

**DOI:** 10.1155/2016/7405930

**Published:** 2016-08-04

**Authors:** Beatrice P. Concepcion, Rachel C. Forbes, Aihua Bian, Heidi M. Schaefer

**Affiliations:** ^1^Department of Medicine, Division of Nephrology and Hypertension, Vanderbilt University Medical Center, Nashville, TN 37232, USA; ^2^Vanderbilt Transplant Center, Vanderbilt University Medical Center, Nashville, TN 37232, USA; ^3^Department of Surgery, Division of Kidney and Pancreas Transplantation, Vanderbilt University Medical Center, Nashville, TN 37232, USA; ^4^Department of Biostatistics, Vanderbilt School of Medicine, Nashville, TN 37232, USA

## Abstract

*Background*. The kidney transplant evaluation process for older candidates is complex due to the presence of multiple comorbid conditions.* Methods*. We retrospectively reviewed patients ≥60 years referred to our center for kidney transplantation over a 3-year period. Variables were collected to identify reasons for patients being turned down and to determine the number of unnecessary tests performed. Statistical analysis was performed to estimate the association between clinical predictors and listing status.* Results*. 345 patients were included in the statistical analysis. 31.6% of patients were turned down: 44% due to coronary artery disease (CAD), peripheral vascular disease (PVD), or both. After adjustment for patient demographics and comorbid conditions, history of CAD, PVD, or both (OR = 1.75, 95% CI (1.20, 2.56), *p* = 0.004), chronic obstructive pulmonary disease (OR = 8.75, 95% CI (2.81, 27.20), *p* = 0.0002), and cancer (OR 2.59, 95% CI (1.18, 5.67), *p* = 0.02) were associated with a higher risk of being turned down. 14.8% of patients underwent unnecessary basic testing and 9.6% underwent unnecessary supplementary testing with the charges over a 3-year period estimated at $304,337.* Conclusion*. A significant number of older candidates are deemed unacceptable for kidney transplantation with primary reasons cited as CAD and PVD. The overall burden of unnecessary testing is substantial and potentially avoidable.

## 1. Introduction

The number of older patients with renal failure being referred for kidney transplantation is increasing. Due to the high burden of comorbid conditions in this patient population, the transplant evaluation process can be quite complex and usually entails multiple diagnostic testing to determine if a patient is medically suitable for kidney transplantation [1]. Very little is known about reasons for patient exclusion among the elderly and how the process for determining suitability for transplant is carried out.

**Figure 1:**
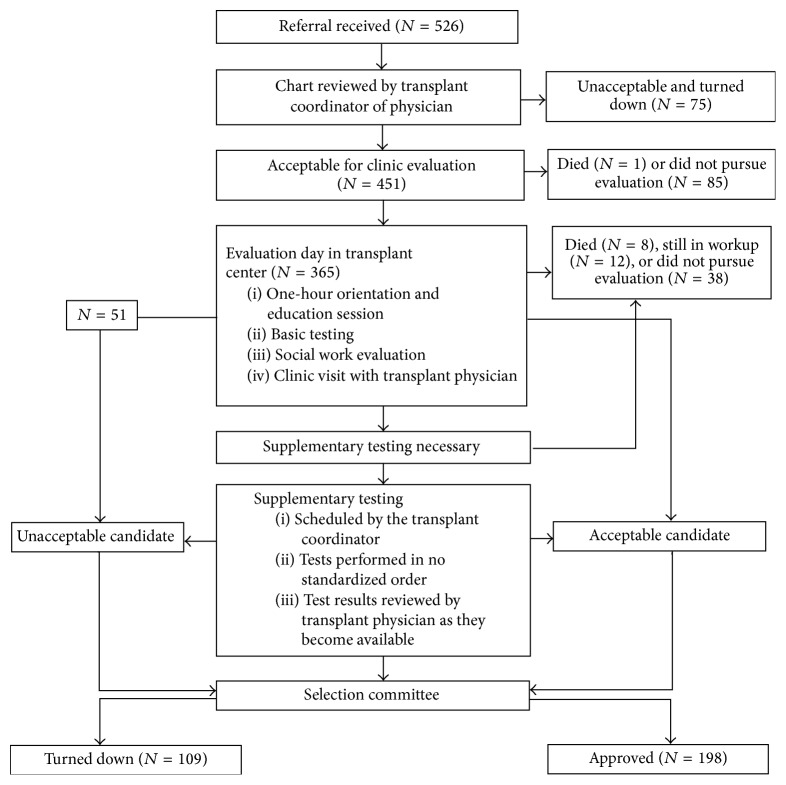
Overview of the kidney transplant evaluation process in our center.

At our center, all patients who are evaluated in clinic undergo basic diagnostic testing (Table 1). The majority of patients who make it through the evaluation day and basic testing undergo supplementary testing (Table 1) as ordered by the physician. There is no established algorithm that determines the order in which requested diagnostic tests are performed. There may be potential for improving the efficiency and cost-effectiveness of this process (Figure 1).

The objectives of this study are (i) to determine the outcomes of the evaluation process in older patients focusing on the reason patients are turned down for kidney transplantation, (ii) to identify clinical characteristics associated with an increased risk of being turned down, (iii) to quantify unnecessary testing that is performed and its related cost, and (iv) to identify potential modifications to our process that may improve cost-effectiveness.

## 2. Materials and Methods

We performed a retrospective chart review of all patients ≥60 years referred to our center for kidney transplantation from January 2006 to December 2009. Patient characteristics collected included age, sex, race, cause of kidney disease, dialysis vintage, body mass index (BMI), history of diabetes mellitus (DM), coronary artery disease (CAD), peripheral vascular disease (PVD), cerebrovascular accident, chronic obstructive pulmonary disease (COPD), pulmonary hypertension, cancer, liver disease, and dementia. These patient characteristics were collected as potential clinical predictors of the outcome in question. Also collected were the outcome of the evaluation process (approved, turned down, died during workup, and did not pursue/continue workup) and the reasons cited for being turned down (CAD, PVD, pulmonary hypertension, cancer, liver disease, lung disease, obesity, dementia/poor cognition, poor physical function, poor social support, noncompliance, and others). We also noted the supplementary diagnostic testing that was performed after clinic evaluation as requested by the evaluating physician. These tests included left and right heart catheterization, cardiac stress test, 2D-echocardiogram, carotid duplex ultrasound, computed tomography (CT) of abdomen/pelvis with/without angiography, pulmonary function test, voiding cystourethrogram, hypercoagulable profile, and liver biopsy.

Descriptive statistics are presented as medians and interquartile range (IQR) for continuous variables and as frequencies and percentages for categorical variables. The differences between patients' approval statuses were compared using Wilcoxon rank-sum test for continuous variables and Pearson's chi-square test for categorical variables.

The independent association of clinical risk factors with approval status was assessed using a multivariable logistic regression model. Covariates for adjustment were chosen* a priori* based on factors and included age, sex, duration of dialysis, CAD or PVD or both, COPD, cause of renal failure, and cancer. Age was included into the model with restricted cubic splines to capture nonlinear relationships.

Statistical analysis was performed using R Version 3.2.1 (http://www.r-project.org) and 2-sided *p* values < 0.05 were considered statistically significant.

The number of unnecessary diagnostic tests performed was determined by reviewing each patient's medical record. An unnecessary diagnostic test was defined as a test whose result was not a factor in the decision to turn down or proceed with additional testing in a kidney transplant candidate. Diagnostic tests related to age-appropriate cancer screening were excluded. The charges in US dollars per diagnostic test were calculated based on our center's charges in 2015. The total estimated charges of unnecessary tests over a 3-year period were then calculated.

This study was approved by our center's institutional review board (IRB number 110359).

## 3. Results

Our study included 526 patients (Table 2 and Figure 1) who were ≥60 years and referred to our center from 2006 to 2009. All patients underwent initial screening via chart review by a transplant coordinator or physician. There were 85 (16.1%) patients who were deemed acceptable but did not pursue further transplant evaluation. There were 75 (14.2%) patients who were deemed unacceptable and were not evaluated in clinic. The reasons for being turned down based on chart review were CAD (23/75), PVD (15/75), cancer (10/75), multiple medical problems (9/75), and insurance issues (6/75). One patient died prior to being evaluated in clinic.

There were 365 patients (Table 2 and Figure 1) who presented to our center for transplant evaluation and were seen in clinic. Twenty patients who died (*N* = 8) during their workup or had not completed testing at the conclusion of the study (*N* = 12) were excluded, leaving a total of 345 patients for analysis. Of these 345 patients, 198 were approved for kidney transplantation, 38 opted not to proceed further (at varying stages in their evaluation process), and 109 were turned down. The top reasons cited for being turned down (with multiple reasons cited for some patients) were PVD (27), CAD (26), obesity (10), poor physical function/frailty (10), poor social support or noncompliance (9), estimated glomerular filtration rate above 20 mL/min/m^2^ (9), lung disease (8), cancer (8), and multiple medical problems (5). CAD, PVD, or both were cited as a reason for being turned down in 48/109 (44%) of all patients turned down. Results of cardiac catheterization (17/26 patients) or CT angiography of abdomen/pelvis (25/27 patients) were the basis for this decision in majority of patients. Specifically, cardiac catheterization findings showing a severe burden of disease such as 3-vessel disease or single vessel disease not amenable to intervention with corresponding ischemia on stress testing usually resulted in a patient being turned down. CT angiography findings of severe calcification with no surgical targets or presence of stenosis or indwelling stents usually resulted in a patient being turned down.

**Figure 2:**
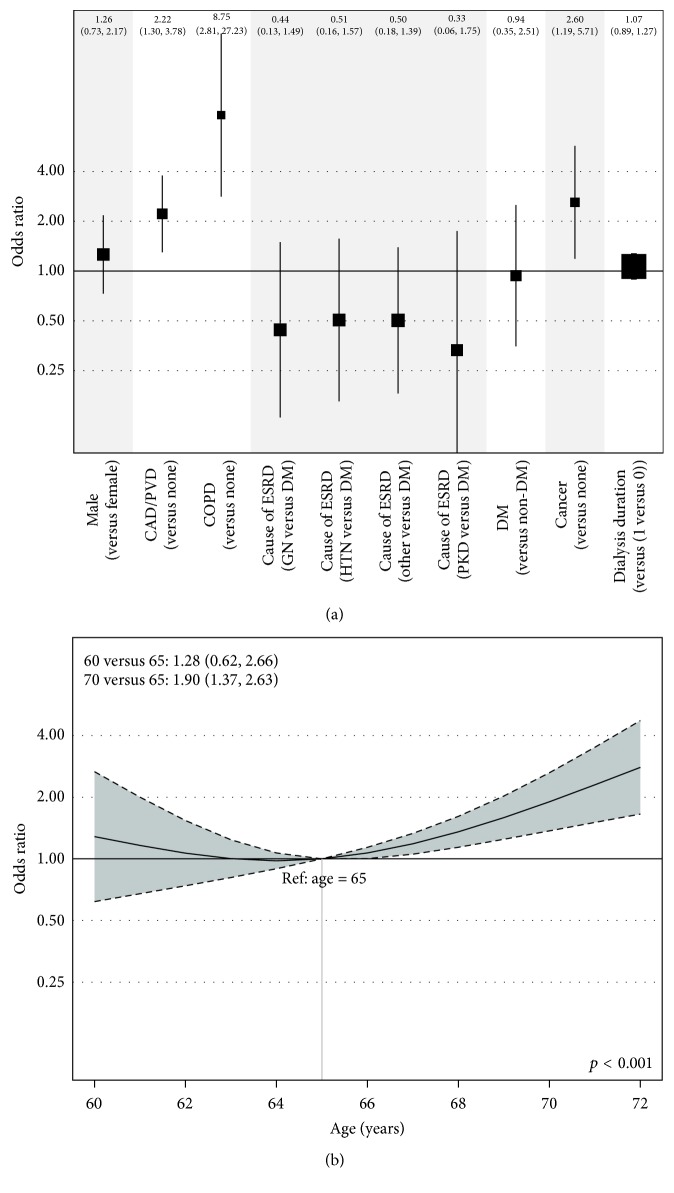
The logistic regression model estimates from the cohorts with 345 patients. (a) Selected clinical characteristics and the odds ratio for being turned down. (b) Patient age at time of referral and the odds ratio for being turned down.

The clinical characteristics at time of referral of the 345 patients who completed the transplant workup are shown in Table 3. Compared with patients who were not turned down (approved or did not proceed with additional workup) during the study period, patients who were turned down were more likely to be older (66 years [63, 70] versus 64 years [62, 67], *p* < 0.001), have diabetes (59% versus 47%, *p* = 0.04), and have CAD, PVD, or both (60% versus 33%, *p* < 0.001). There was no significant difference in dialysis duration as most patients in both groups were either not yet on dialysis or had been on dialysis for less than a year at time of referral for transplant.  Figure 2(a) shows the results from the primary analysis using a logistic regression model of the approval status. The odds ratio values represent the increased odds of being turned down for each variable as compared with the reference group or values. After adjustment for age, gender, and duration of dialysis, the following clinical characteristics were significantly associated with being turned down: history of CAD, PVD, or both (OR = 1.75, 95% CI (1.20, 2.56), *p* = 0.004), history of COPD (OR = 8.75, 95% CI (2.81, 27.20), *p* = 0.0002), and history of cancer (OR 2.59, 95% CI (1.18, 5.67), *p* = 0.02). Dialysis duration was not statistically significantly associated with approval status (*p* = 0.48).  Figure 2(b) shows that the impact of age on the approval status was nonlinear. While the effect of age on outcome was not statistically significant for patients younger than 65 years, patients older than 65 had higher odds of being turned down (*p* < 0.001).

All 345 patients included in our analysis underwent basic testing (Figure 1). There were 51 patients who were turned down after history and physical examination was obtained in the clinic evaluation and therefore underwent unnecessary basic testing (Table 4). Using current charges in our center, the estimated charges related to this amounted to $137,343 and per patient was $2,693 (Table 1). In addition, 33 patients underwent unnecessary supplementary testing (Table 4) of whom 24 were ultimately turned down due to CAD, PVD, or both. The charges related to this amounted to $166,994 (Table 1). The total charges of unnecessary testing in older kidney transplant candidates performed in our center over a 3-year period amounted to $304,337 (Table 1).

## 4. Discussion

A significant number of older patients referred for kidney transplantation were deemed unsuitable candidates after undergoing our center's transplant evaluation process (Table 4). CAD and PVD were the main reasons for patients being turned down. This is consistent with the findings of Holley et al. where heart disease was cited as a reason for exclusion in 38% of patients who were medically unsuitable [2]. Our finding is not surprising as cardiovascular disease is the leading cause of morbidity and mortality in transplant recipients [3]; hence probably the organ system is most closely scrutinized during a patient's medical evaluation. In our study, we found that majority of patients turned down due to CAD or PVD underwent a left heart catheterization or CT angiography, respectively, and the findings on these tests formed the basis for being turned down. The appropriate cardiovascular testing for patients undergoing transplant evaluation remains controversial and center-specific [4]. In our center, patients who have known CAD, who have longstanding DM greater than 20 years, or who are 70 years or older are required to undergo left heart catheterization if one had not been performed within the last 3–5 years. An abnormal cardiac stress test also necessitates subsequent catheterization. The suboptimal sensitivity of both dobutamine stress echocardiograms and nuclear stress testing especially in diabetic ESRD patients, along with the concomitant high prevalence of CAD in this patient population, is the rationale behind this approach. In patients with a history of or are suspected to have peripheral vascular disease based on exam findings during the evaluation, CT angiography is the usual modality by which our center evaluates the suitability of iliac vessels for kidney transplantation. This is preferred over arterial duplex ultrasound due to the better depiction of arterial anatomy by CT.

Interestingly, in addition to CAD and PVD, we found that a history of COPD and cancer also placed a candidate at a much higher risk of being turned down. These patients however numbered much less than those with cardiovascular disease. Although this was not specifically investigated in the study, a possible explanation for the high-risk for being turned down in patients with a history of cancer is they may have required additional waiting time based on accepted guidelines [5]. In contrast to Holley et al. where 10% of all patients were excluded due to obesity, only 2.9% of patients in our study were turned down for this reason. This may be partly due to the higher BMI cutoff employed at our center wherein patients with BMI between 40 and 45 are considered on an individual basis.

A striking finding in this study is the number of unnecessary testing that was performed and its related estimated cost. We routinely obtain blood testing, chest radiographs, and electrocardiograms for all patients who come to our center for transplant evaluation. In this study we found that 14.8% of older candidates were deemed unsuitable for transplant based on history and physical examination alone. These patients underwent unnecessary basic testing which amounted to estimated charges of $137,343 or $2,693 per patient. A change to our process that would result in avoiding these unnecessary charges would be to defer basic testing until a patient is evaluated by a physician in clinic. This would involve changing the current schedule that a patient follows during the evaluation day in our center; that is, patient would see the physician first before obtaining basic testing. If a patient is thought to be unsuitable or requires further review of records, then basic testing would not be obtained during the evaluation day.

The majority of our patients who make it through the evaluation day and basic testing require some form of supplementary testing based on the presence of certain comorbid conditions. For example, patients with COPD undergo pulmonary function testing or those with a history of an ischemic stroke undergo carotid duplex ultrasound. The required supplementary tests are usually determined after clinic evaluation. The patient's transplant coordinator oversees the patient's subsequent workup by scheduling required testing and communicating with the physician regarding results. There is no established algorithm that determines the order in which requested tests are performed. In this study, 33 patients underwent unnecessary supplementary testing with the estimated charges amounting to $166,994. As an example, a patient with a history of CAD and ischemic stroke was requested to undergo left heart catheterization and carotid duplex ultrasound. The duplex ultrasound was performed before the catheterization and the patient was ultimately turned down due to severe CAD. The carotid duplex ultrasound, which is charged at $677, was unnecessary and would not have been performed if the findings on catheterization had already been known. We learned in this study that 44% of older patients were turned down in our center due to CAD or PVD and that, in the majority of cases, a left heart catheterization or CT angiography was the basis for the decision. A change to our process that would improve cost-effectiveness would be to establish an algorithm for supplementary testing wherein the cardiovascular workup such as stress tests, left heart catheterizations, and CT angiograms is obtained prior to pursuing other testing. If the cardiovascular workup deems a candidate unsuitable for transplant, then there would be no need to proceed with further testing.

In an age of rising health care costs, limited resources, and professional time, there is certainly an impetus for improving cost-effectiveness in the transplant evaluation process. As more and more elderly and complex patients are referred for kidney transplantation, cost will presumably increase. Our study showed that, by examining our center's evaluation process and gaining a better understanding of why patients are turned down, certain potential cost-saving measures can be identified and initiated. Importantly, the burden of unnecessary testing likely goes beyond the financial cost that is depicted in this study. The undue time spent by clinicians, coordinators, ancillary staff, and patients leads to wasted productivity. Patients who undergo unnecessary testing also take on associated risks related to these tests, for example, undue radiation exposure. Finally, the transplant center itself as an organization may experience a reduction in throughput and efficiency.

Our study has several limitations. First, our study cohort is from 2006 to 2009 and although our evaluation process and written criteria for transplant eligibility have not changed, it is possible that our transplant center's pattern of approving or turning down candidates may have changed over time; for example, our center may be approving an increased volume of older and sicker candidates currently compared to 2009. Second, our study involves only our transplant center and because every center's evaluation process is unique, our study's findings may not necessarily be applicable to other centers whose processes are much different than ours. This study however highlights a need for every center to examine its own processes. Finally, in analyzing the cost-effectiveness of our process, we used our center's charges and not actual patients' costs. As charges are usually inflated, the true cost-estimate of the unnecessary testing performed may be significantly lower than what we calculated.

There is limited data on this issue and, to our knowledge, there is only one other study similar to ours [2]. Other studies have similarly examined their respective centers' evaluation process with the goal of improving efficiency, effectiveness, and quality [6, 7]. In this study, we were able to examine a robust number of older patients undergoing transplant evaluation in a large academic center which performs approximately 150 to 200 kidney transplants annually. Further studies focusing on improving cost-effectiveness of the transplant evaluation process are needed.

## 5. Conclusions

In conclusion, approximately 1/3 of older patients are deemed unacceptable candidates for kidney transplantation in our center, even after screening via chart review prior to a clinic evaluation. A history of CAD, PVD, COPD, or cancer places a candidate at an increased risk for being turned down. Ultimately, CAD and PVD are the primary reasons cited for being turned down. Additionally, among patients who are evaluated in clinic and are ultimately turned down for transplant, 77% undergo unnecessary testing. The estimated charges of unnecessary testing are significant. In addition to this financial burden, nonquantifiable costs of unnecessary testing may include undue time spent by providers and patients, unwarranted risks associated with tests that are undertaken by patients, and reduction in throughput in a transplant center. Strategies to improve cost-effectiveness may include deferring basic testing for patients who are high-risk for being turned down until after they are evaluated by a physician in clinic. In patients with a history of CAD or PVD, completing the cardiovascular workup and, if clinically indicated, obtaining a cardiac catheterization and CT angiography should be prioritized over other supplementary diagnostic tests.

## Figures and Tables

**Table 1:** Estimated charges of “basic” and “supplementary” diagnostic tests.

Diagnostic tests	Estimated charges in USD per test	Number of patients who underwent unnecessary testing	Estimated charges in USD per test multiplied by number of patients
*Basic testing*			
Complete blood count	81	51	4,131
Comprehensive metabolic panel	170	51	8,670
Coagulation profile (PT^a^, PTT^b^)	141	51	7,191
Blood typing	97	51	4,947
Human leukocyte antigen typing	500	51	25,500
Panel reactive antibodies	100	51	5,100
Rapid plasma reagin	59	51	3,009
Electrocardiogram	225	51	11,475
Chest radiograph	312	51	15,912
Viral serologies (hepatitis B surface Ag^c^, hepatitis B core Ab^d^, hepatitis B surface Ab, HCV^e^ Ab, HIV^f^ Ab, CMV^g^ IgG^h^/IgM^i^, EBV^j^ IgG^h^/IgM^i^)	1,008	51	51,408

*Total*	*2,693*	51	*137,343*

*Supplementary testing*			
Dobutamine stress echocardiogram	1,747	10	17,470
Nuclear (regadenoson) stress test	3,663	1	3,663
2D-echocardiogram with Doppler	3,091	2	6,182
Left heart catheterization	9,500	9	85,500
Carotid arterial Doppler	677	10	6,770
CT^k^ angiography of abdomen/pelvis	5,010	7	35,070
CT^k^ abdomen/pelvis with/without contrast	5,232	1	5,232
Pulmonary function test	525	4	2,100
Lower extremity arterial Doppler	604	2	1,208
CT^k^ chest with/without contrast	2,624	1	2,624
24-hour Holter monitoring	711	1	711
Voiding cystourethrogram	464	1	464

*Total*			*166,994*

^a^PT, prothrombin time; ^b^PTT, partial thromboplastin time; ^c^Ag, antigen; ^d^Ab, antibody; ^e^HCV, hepatitis C virus; ^f^HIV, human immunodeficiency virus; ^g^CMV, cytomegalovirus; ^h^IgG, immunoglobulin G; ^i^IgM, immunoglobulin M; ^j^EBV, Epstein-Barr virus; ^k^CT, computed tomography.

**Table 2:** Summary of patients and their referral/evaluation outcomes.

Patient outcomes after referral/evaluation	All patients referred for transplant *N* = 526		All patients evaluated in clinic *N* = 365	
	Number of patients	% of total (526)	Number of patients	% of total (365)
Accepted for transplant	198	38%	198	54%
Turned down for transplant	184	35%	109	30%
Voluntary quit from transplant evaluation process	123	23%	38	11%
Died	9	2%	8	2%
Still in workup	12	2%	12	3%

Totals	526	100%	365	100%

**Table 3:** Clinical characteristics at time of referral of patients by approval status.

	Patients not turned down (*N* = 236)	Patients turned down (*N* = 109)	All patients (*N* = 345)	*p* value
Age at referral, y	64.0 [62.0, 67.0]	66.0 [63.0, 70.0]	64.0 [62.0, 68.0]	<0.001
Female (%)	100 (42)	37 (34)	137 (40)	0.14
Race (%)				0.47
(i) African American	55 (23)	27 (26)	82 (24)	
(ii) Caucasian	171 (73)	75 (73)	246 (73)	
(iii) Hispanic	5 (2)	1 (1)	6 (2)	
(iv) Others	4 (2)	0 (0)	4 (1)	
Cause of renal failure				0.052
(i) DM^a^ (%)	88 (37)	57 (52)	145 (42)	
(ii) HTN^b^ (%)	46 (19)	19 (17)	65 (19)	
(iii) PKD^c^ (%)	19 (8)	3 (3)	22 (6)	
(iv) GN^d^ (%)	29 (12)	8 (7)	37 (11)	
(v) Other (%)	54 (23)	22 (20)	76 (22)	
Duration of dialysis, y	0 [0, 1]	0 [0, 2]	0 [0, 1]	0.05
BMI^e^, kg/m^2^	28.0 [24.8, 33.2]	28.3 [24.3, 32.6]	28.0 [24.8, 33.0]	0.90
DM^a^ (%)	111 (47)	64 (59)	175 (51)	0.044
CAD^f^ (%)	66 (28)	53 (49)	119 (34)	<0.001
PVD^g^ (%)	23 (10)	31 (28)	54 (16)	<0.001
CVA^h^ (%)	37 (16)	21 (19)	58 (17)	0.42
COPD^i^ (%)	5 (2)	15 (14)	20 (6)	<0.001
Pulmonary HTN^b^ (%)	0 (0)	2 (2)	3 (1)	0.19
Cancer (%)	17 (7)	18 (17)	35 (10)	0.008
Liver disease (%)	7 (3)	5 (5)	12 (3)	0.45

Data presented as median [interquartile range] or frequencies with proportions (%). *p* values from Wilcoxon rank-sum tests for continuous variables and Pearson's chi-square tests for categorical variables.

^a^DM, diabetes mellitus; ^b^HTN, hypertension; ^c^PKD, polycystic kidney disease; ^d^GN, glomerulonephritis; ^e^BMI, body mass index; ^f^CAD, coronary artery disease; ^g^PVD, peripheral vascular disease; ^h^CVA, cerebrovascular accident; ^i^COPD, chronic obstructive pulmonary disease.

**Table 4:** Summary of all patients turned down for transplant and unnecessary testing.

Timing of patient being turned down	Number of patients turned down	Number of patients with unnecessary testing	Charges associated with unnecessary testing (USD)	All patients referred for transplant and turned down (*N* = 184)^a^	All patients evaluated in clinic and turned down (*N* = 109)^b^
Chart review	75	0	0		
H&P^c^ with physician	51	0	0		
Basic diagnostic testing		51	137,343		
Supplementary diagnostic testing	58	33	304,337		

Totals	184	84	

Overall % of patients with unnecessary testing and charges				46%	77%

^a^Patients who were turned down after chart review; ^b^only patients who were evaluated in clinic; ^c^H&P, history and physical examination.
